# Personal Recollections of a Dentist of the Early Days

**Published:** 1886-01

**Authors:** L. W. Bristol

**Affiliations:** Lockport, N. Y.


### PERSONAL RECOLLECTIONS OF A DENTIST OF THE EARLY DAYS.

BY DR. L. W. BRISTOL, LOCKPORT, N. Y.

READ BEFORE A UNION MEETING OF THE SEVENTH AND EIGHTH DISTRICT DENTAL SOCIETIES OF THE STATE OF NEW YORK, HELD IN BUFFALO, OCTOBER 27 AND 28, 1885.

PUBLISHED AT THE REQUEST OF THE SOCIETIES.

About the year 1837 or 1838, there was a dentist in practice in the City of Buffalo, by the name of Bigelow. He used to sally out into the neighboring towns and hamlets, and once dropped down upon the village of Lockport. His fees, in those early days, would compare with and equal the high charges of some of our eastern practitioners of the present day. He spent about two hours cleaning and filling the teeth of the wife of a celebrated lawyer in Lockport, Mr. C——. His charges were fifty dollars. Mr. C—— thought that was a most extravagant price, and said so. Bigelow replied: "We professional men must be liberally paid for our services," and would make no discount. The lawyer paid the bill and took a receipt. After Bigelow had left the village, the lawyer happened to remember that, in a business transaction with some parties in Buffalo, he had taken a mortgage on some real estate there, given by a Dr. Bigelow. He overhauled his mortgages and found this was his man, and that the mortgage was long past due. He set about an immediate foreclosure of the same, in the most expensive way known to the legal profession. Bigelow hurried down and expostulated. He thought the fee demanded was extravagant and extortionate, but Lawyer C—— replied: "We professional men must be very liberally paid for our services."

Bigelow once did some work for a lady who said that her husband was a great showman, and he thought that his chance had come to make a strike. He improved the opportunity and charged a big price, which was paid, and a receipt taken. In a short time the man opened a museum, and in advertising his curiosities, after enumerating a lot of rare things, he said: "The most rare and extravagant thing in the whole collection is a 'Dentist's bill receipted.'" And there, sure enough, in a big tin frame, suspended by a chain to a post, in the most conspicuous place in the museum, was Bigelow's bill for dental services for wife. It was a wet blanket for Bigelow, and annoyed him excessively. He finally got a friend to go and buy the thing, paying about the same for it that he had charged for dental services.

About 1844, the late Dr. O. W. May had made a gold plate with six teeth, for a man by the name of Easton. The gums had settled away, the clasps had broken and the plate was loose, and it dropped down when in conversation. Easton had become offended with May, and took great delight in showing the work. He would say, "There is a specimen of Dr. May's work," and then would wobble them up and down. He would not let May touch them, and they were really a very bad advertisement. May said to me one day, "Doctor, I will give you twenty dollars if you will get those teeth out and make him a set." I agreed to try. A few days after I happened to meet the old gentlemen, and he showed me how the teeth wobbled. I said to him, "They are not much comfort to you?" "No," he replied; "I stick to them to annoy May, but I am tired of the fun. What will you charge to make me a set that will fit?" I agreed to make them for the old plate and twenty dollars. He came to my office. I took an impression and made him a set that proved satisfactory to him, and he paid the bill. In a couple of weeks I met Dr. May, who said: "I see you have got the old teeth out of Easton's head; here is your twenty dollars." I took the money and said to him, "Here is your old gold plate and teeth; I guess all parties are perfectly satisfied; at least I am." I think that was the only time I ever received a double fee for making a set of teeth.

Dentists in those early days used to visit the surrounding towns and villages a great deal more than they do now. I remember visiting my old stamping ground, Lewiston, and was told that another dentist from an eastern city had come there the day before, had taken rooms and was going to open an office. I got up early the next morning, and took a stroll down to the Niagara River, and as I was returning I saw a man tacking a card on Bartlet's store door. Just then a well-known citizen came along, saw the card, shook the door, and yelled out: "Get up, Bartlet; the sheriff is advertising your premises." The new dentist, Dr. M., replied: "That is not a sheriff's notice; it is a dentist's advertisement." At that the man seized and shook the door more violently than before, crying out at the top of his voice: "Get up, get up, Bartlet, the dentist is advertising your whole real estate." Dr. M. stole away, looking sheepish. In the afternoon I called at his rooms and found him packing up. He said that was no place for him; if he stuck up a card they said he was advertising their property at sheriff's sale. By the way, that dentist has since made his mark in the profession, and is a splendid operator, and an ornament to the profession. He does not as easily get discouraged at a joke now.

There is not now as much ignorance and superstition among the mass of people, relative to the teeth or practice of medicine, as there was forty or fifty years ago. It was not an uncommon request then for our patients to ask us not to touch their teeth with our fingers after extraction, but to roll them up in paper for them. I sometimes had the curiosity to ask what was to be done with them, and was told they were going to bore a hole in a sweet apple tree, put the tooth in and plug it up. Others were going to take the tooth and go out to the woods, turn their backs, take three steps, and throw the tooth back over their heads and say: "Good-bye to toothache." Others were going to burn them, and scatter the ashes to the four quarters of the earth, and in that way get rid of the toothache, but I judge these were of little effect, for I have extracted teeth subsequently for the same persons.

The old superstitions have not entirely died out, and are practiced yet, as witness the carrying of a small potato or a horse-chestnut in the pocket to ward off the rheumatism, or cutting the finger nails only on Friday, to prevent headache, or avoiding the beginning of a journey, or the building of a house, or the commencement of a set of teeth on Friday.

We used to have a great deal of trouble with our porcelain blocks of teeth; they were sure to break, if dropped while carelessly handled. I remember a block of upper teeth put in for the Rev. B—, in one of the neighboring villages. He was in the midst of a morning sermon, and was describing Saul on the road to Damascus. In quoting the passage, "Saul, Saul, why persecutest thou me," he leaned over the pulpit, when his teeth dropped out and fell on the table below and broke in three pieces. He looked down at the wreck and exclaimed, "My friendth, you thee the fixth I'm in—Amen."

He went home and wrote me of his trouble, desiring me to come to him immediately. I did so the next day, and made him a new set. He was always lecturing me for not being a member of a church, and while at dinner opened on me again. I remarked that it came with ill grace for him to reprove me when he was guilty of transacting and writing business letters on the Sabbath. He denied the charge, and I produced his letter to me, dated on Sunday. His wife remarked, "Now, Father, you had better dry up."

The Rev. Glezen Fillmore, than whom no purer, better man lived, was pastor of a Methodist church then located on Walnut Street. He wore a set of teeth on silver plates, made by Dr. Hains, of Rochester. The plate had cracked between the central incisors, had been soldered, but was giving out again. There was a revival, and at the morning service the church was well filled. I happened to be in attendance, as usual. He was saying, "The spider's most attenuated web is cable when compared to the thread on which hangs the life of poor mortality." Before he got half through the sentence the crack in his plate opened, the solder came off and the words whistled out as though he had a toy tin whistle in his mouth. Everybody was convulsed with laughter. He stopped and sucked away at them, but there was no improvement. "We will turn this morning service into a prayer meeting," said he; "Bro. P—, will you lead in prayer?" He retired to a back room, and directly one of the members tapped me on the shoulder, and said that Mr. Fillmore wanted to see me. When I arrived in his presence he declared that I must accompany him to my office and mend the teeth. I went with him, took the teeth in hand, and he took my bible—I had one in my office, as all well regulated dentists should—and studied out another sermon. I got the teeth mended and in his mouth just as the bells were ringing for evening service. "Now come over and I will give you a first-rate sermon," said he. I did so, and was well pleased that I had done a creditable job in dentistry, and I had no idea that so good a sermon could emanate from my office. Some members of his congregation afterwards suggested that he had better visit my office every Sabbath morning.

The most remarkable case of wearing a dilapidated set of teeth I ever knew, was that of a maiden lady, Miss F——. Dr. Thos. Harrison had made her an upper and under set on porcelain. By some means or other both plates were broken in the center. When I saw her she was wearing the teeth. She would take all four pieces in her hand, put them in her mouth, and with her tongue adjust them to their proper place in less time than I can describe it. It was amusing to hear her talk, as the blocks would rattle and knock together. The ends were worn smooth and rounded off. When she died the teeth were placed in her mouth and buried with her. Don't let any one desecrate her grave in hopes of securing a valuable dental structure.

A few years since, I made a partial denture for a Mrs. L—., a German lady. Every little while she would bring them to me with the left lateral incisor broken. I could not account for it, as she had no antagonizing teeth on the under jaw. I said, "Mrs. L—, I cannot understand this. How do you break that tooth so often?" She hesitated a little, but finally said: "Well, I let Susan wear them when she goes to a party or dance, and she is the one that breaks that tooth off." That explained the mystery. I had made a partnership set of teeth, to be worn by the mother and broken by the daughter. This was the only case I ever knew in which a partial set of teeth could be used by two persons. I finally made a set for the daughter, and had no further trouble with repairing the mother's teeth.

NOTE.—Th's paper is a continuation of the one published in this journal for December, 1884. Dr. Bristol is now the Father of the Profession in Western New York. He is, and has always been, an original character, and much of the interest that is felt in his personal narrations is due to his inimitable manner of relating them. Unfortunately, this is quite lost when they are in print. He is full of anecdotes of the early days, and the societies before whom this and the paper of last year were read, by unanimous vote requested him each year to furnish a chapter of his recollections. In promising compliance he said that he belonged to another generation. His old friends and former companions had passed away. Whitney, Hayes, Snow and others, were no more, and he felt

——like one who treads alone
Some banquet hall deserted;
Whose lights are fled, whose garlands dead,
And all but he departed!

—EDITOR.

